# Endocrine and Neuroendocrine Tumors: A Special Issue

**DOI:** 10.3390/cancers14204994

**Published:** 2022-10-12

**Authors:** Alfredo Berruti, Vito Amoroso, Nicola Fazio

**Affiliations:** 1 Medical Oncology Unit, Department of Medical and Surgical Specialties, Radiological Sciences and Public Health, University of Brescia, ASST Spedali Civili of Brescia, 25123 Brescia, Italy; 2 Division of Gastrointestinal Medical Oncology and Neuroendocrine Tumors, European Institute of Oncology, IEO, IRCCS, 20132 Milan, Italy

Endocrine and neuroendocrine tumors (NETs) represent a group of heterogeneous malignancies that have endocrine cell onset as a common denominator. They are relatively rare and may be classified within inherited syndromes. The prognoses are various and mainly related to tumor stage at diagnosis and tumor grade. The treatment of patients with these malignancies is often challenging and therefore requires a multidisciplinary approach. In recent years, we have witnessed an improvement in the biological and pathogenetic knowledge of these diseases, and new effective drugs have been identified [1,2].

This Special Issue, which comprises 12 papers (seven original articles and five reviews), addresses various aspects concerning the state of the art and future perspectives in the field of clinical and translational research of endocrine and neuroendocrine tumors.

As regards the original contributions, adrenocortical carcinoma (ACC) is an extremely rare and challenging malignancy [3]. The paper by Sbiera et al. [4] addressed an important topic: the epithelial to mesenchymal transition (EMT) as a potential mechanism associated with metastasis in adrenocortical carcinoma (ACC). The authors studied EMT in tissues from 138 ACC, 29 adrenocortical adenomas and three normal adrenal glands. The results showed that both normal and neoplastic adrenocortical tissues showed no expression of epithelial markers, but strongly expressed mesenchymal markers. The authors concluded that there is no indication of EMT in ACC as all adrenocortical tissues showed no expression of epithelial markers and exhibited closer similarity to mesenchymal tissues. However, they observed that the EMT marker SLUG seems to be associated with a more aggressive phenotype.

Multiple Endocrine Neoplasia-2 (MEN-2) has a very high penetrance in medullary thyroid carcinoma (MTC) patients, although with intra- and inter-familial variability. The paper by Prete et al. [5] reported results of a large prospective study that involved 189 gene carrier patients, of whom 67 were admitted to immediate thyroid surgery and 122 were followed-up and were sent to surgery only if they met pre-defined clinical and biochemical characteristics. In the follow-up group, only 22 patients underwent surgery, while 100 could spare their thyroid at least at the last examination visit. This important study demonstrated that a patient-centered surveillance approach permits postponing thyroid surgery in children until adolescence/adulthood.

Although peptide receptor radionuclide therapy (PRRT) is an effective therapeutic option in patients with metastatic NET, this treatment modality is inefficacious in about 15–30% of cases. The paper by Durmo et al. [6] was designed to retrospectively identify biomarkers able to predict responses to PRRT in metastatic NET patients with different primary malignancies. The results found that a high baseline tumor volume was the only parameter negatively associated with the disease response to PRRT and patient survival.

The study by Lamarca et al. [7] explored the feasibility of assessing circulating tumor DNA (ctDNA) in a cohort of 15 patients with advanced well-differentiated NETs. A cohort of 30 patients with non-WD NETs was utilized for comparative purposes only. In this study, mutation-based ctDNA analysis, although feasible (with a non-evaluable sample rate of 27.8%), was of limited clinical utility.

Few cellular and patient-derived xenograft (PDX) models are available for testing new therapies and studying the heterogeneous nature of neuroendocrine neoplasms (NENs). Tran et al. [8] described the establishment and characterization of two novel neuroendocrine carcinoma (NEC) cellular and PDX models (NEC913 and NEC1452). NEC913 PDX tumors expressed somatostatin receptor 2 (SSTR2), whereas NEC1452 PDX tumors were SSTR2-negative. As a proof-of-concept study, the authors demonstrated how these PDX models can be used for peptide imaging experiments targeting SSTR2 using fluorescently labeled octreotides.

Several societies have issued guidelines for the diagnosis and treatment of NETs; however, there are still areas of controversy for which there is limited guidance. A group of experts met to formulate 14 statements regarding controversial issues relative to the diagnosis, treatment, and follow-up of NENs, which are presented in the paper by Bartolomei et al. [9]. The nominal group and estimate–talk–estimate techniques were used. 

Despite the approval of new targeted therapies for pancreatic neuroendocrine tumors (PanNETs) over recent decades, the early identification of resistant tumors remains a major challenge. Cella et al. [10] evaluated a specific soluble angiogenesis panel as a possible predictor of efficacy/resistance to everolimus in patients with PanNETs. This study showed that none of the investigated categories of biomarkers had predictive value for everolimus resistance or efficacy. However, the data suggested that circulating endothelial progenitors might be surrogate biomarkers for angiogenesis activity in PanNETs during everolimus treatment, and their baseline levels might correlate with patients’ survival outcomes.

Among the review papers, Feola et al. [11] performed a systematic literature review to explore the clinicopathological features and the treatment response according to the Ki-67 labeling index cut-off in NECs. A total of 268 NEC patients from eight studies were analyzed. The results showed that NECs with a low Ki-67 labeling index had a better prognosis than the subgroup with higher Ki-67 but worse than G3 NET patients. These results support the notion that NECs are heterogeneous, and their clinical behavior is different from NETs irrespective of the proliferative activity.

Aktypis et al. [12] presented the results of a systematic review and quantitative meta-analysis on the cardiovascular toxicity of biotherapy and molecular targeted therapies currently in use in the management of patients with advanced and/or metastatic NENs. They found that somatostatin analogs and tryptophan hydroxylase inhibitors appeared to be safer than mTOR and tyrosine-kinase inhibitors (TKIs) with regard to cardiovascular toxicity. The authors concluded that special consideration should be given to a patient-tailored approach with anticipated toxicities of targeted treatments for NENs together with the assessment of cardiovascular comorbidities, and early recognition/management of cardiovascular toxicities in order to preserve cardiovascular health and overall quality of life.

Whether immunotherapy is efficacious or not in the management of adrenal cancers is a controversial issue [13]. Jimenez et al. [14] reviewed the current literature to summarize the role of immunotherapy in these rare cancers. The results of clinical trials with immune checkpoint inhibitors (ICIs) for adrenocortical carcinoma or metastatic pheochromocytoma or paraganglioma demonstrated limited benefits; nevertheless, published trials also suggest interesting mechanisms that might enhance clinical responses, including the normalization of tumor vasculature, the modification of the hormonal environment, and vaccination with specific tumor antigens.

Carcinoid crisis is a severe adverse event that may rarely occur in patients with advanced NET. In their review paper, Bardasi et al. [15] discussed its potential etiopathogenetic mechanisms, clinical implications, potential treatments and prophylaxis.

Anaplastic thyroid carcinoma (ATC) is a very aggressive neoplasm and the patient prognosis is dismal [16]. However, a significant survival improvement might be possible in tertiary centers owing to the systematic use of molecular tests for targeted therapies and the integration of fast-track dedicated care pathways. Jannin et al. [17] reviewed the current knowledge on ATC and provided perspectives to improve the management of this deadly disease.

We hope this Special Issue may have responded to the clinical need for up-to-date and in-depth information for the optimal management of patients with NENs.
